# Efficacy and cost-effectiveness of early antiretroviral therapy and partners’ pre-exposure prophylaxis among men who have sex with men in Shenyang, China: a prospective cohort and costing study

**DOI:** 10.1186/s12879-019-4275-x

**Published:** 2019-07-25

**Authors:** Qing-hai Hu, Kathrine Meyers, Jun-jie Xu, Zhen-xing Chu, Jing Zhang, Hai-bo Ding, Xiao-xu Han, Yong-jun Jiang, Wen-qing Geng, Hong Shang

**Affiliations:** 1 NHC Key Laboratory of AIDS Immunology (China Medical University), Department of Laboratory Medicine, The First Affiliated Hospital of China Medical University, Shenyang, 110001 China; 2 Key Laboratory of AIDS Immunology of Liaoning Province, The First Affiliated Hospital of China Medical University, Shenyang, 110001 China; 3 Key Laboratory of AIDS Immunology, Chinese Academy of Medical Sciences, Shenyang, 110001 China; 4 Collaborative Innovation Center for Diagnosis and Treatment of Infectious Diseases, 79 Qingchun Street, Hangzhou, 310003 China; 5 Aaron Diamond AIDS Research Center, The Rockefeller University, New York, NY USA

**Keywords:** Men who have sex with men, Early HIV infection, Antiretroviral therapy, Pre-exposure prophylaxis, Mathematical modeling, Cost-effectiveness analysis

## Abstract

**Background:**

Biomedical interventions such as antiretroviral therapy (ART) and pre-exposure prophylaxis (PrEP) are highly effective for prevention of human immunodeficiency virus (HIV) infection. However, China has not released national PrEP guidelines, and HIV incidence among men who have sex with men (MSM) is unchanged despite substantial scale-up of ART. We evaluated reductions in HIV transmission that may be achieved through early initiation of ART plus partners’ PrEP.

**Methods:**

Six intervention scenarios were evaluated in terms of their impact on HIV transmission and their cost-effectiveness for 36 months post-infection. Three scenarios were based on observed data: non-ART, standard-ART, and early-ART. Another three scenarios were based on observed and hypothetical data: non-ART plus partners’ PrEP, standard-ART plus partners’ PrEP, and early-ART plus partners’ PrEP. The number of onward transmissions was calculated according to viral load and self-reported sexual behaviors, and calibrated by the prevalence and incidence of HIV among Chinese MSM. Cost-effectiveness outcomes were quality-adjusted life-years (QALYs) and cost-utility ratio (CUR).

**Results:**

The estimated number of onward transmissions by every 100 HIV-positive cases 36 months post-infection was 41.83 (95% credible interval: 30.75–57.69) in the non-ART scenario, 7.95 (5.85–10.95) in the early-ART scenario, and 0.79 (0.58–1.09) in the early-ART plus partners’ PrEP scenario. Compared with non-ART, the early-ART and early-ART plus partners’ PrEP scenarios were associated with an 81.0 and 98.1% reduction in HIV transmission, and had a CUR of $12,864/QALY and $16,817/QALY, respectively.

**Conclusions:**

Integrated delivery of early ART and sexual partners’ PrEP could nearly eliminate HIV transmission and reduce costs during the first 36 months of HIV infection. Our results suggest a feasible and cost-effective strategy for reversing the HIV epidemic among MSM in China.

**Electronic supplementary material:**

The online version of this article (10.1186/s12879-019-4275-x) contains supplementary material, which is available to authorized users.

## Background

Several studies have provided crucial evidence that “treatment as prevention” is an effective prevention strategy against human immunodeficiency virus (HIV) infection in men who have sex with men (MSM). The PARTNER and Opposites Attract studies have reported no linked transmissions in HIV serodiscordant male couples after nearly 35,000 acts of unprotected anal intercourse (UAI) in the absence of daily pre-exposure prophylaxis (PrEP) [1, 2]. The 2016 World Health Organization (WHO) guidelines recommend antiretroviral therapy (ART) initiation regardless of the CD4 cell count after diagnosis (“immediate ART”) [3]. By mid-2016, all HIV-infected individuals in China were encouraged to receive immediate ART [4].

However, the substantial global scale-up of ART has not resulted in appreciable reductions in HIV incidence among MSM [5]. In China, annual reported cases of HIV infection among MSM has increased consistently even as treatment coverage has expanded [6, 7]. The pooled HIV incidence has grown rapidly among MSM, from approximately 3.24/100 person-years (PY) in 2005 to 2008 to 5.50/100 PY in 2012 to 2014 [8]. Studies have shown that 38–43% of transmissions occur in the context of early HIV infection (EHI; diagnosis within 6 months after infection) [9, 10], suggesting that those with EHI have higher transmissibility [11–13]. To date, the effectiveness of early ART among MSM to prevent onward transmission has not been reported in China.

PrEP has demonstrated clear reductions in the risk of HIV infection for HIV-negative MSM who adhere to the regimen [14–18]. The latest pooled data from 46 PrEP demonstration projects, involving 10,609 persons and 9,936 PY of follow-up, showed an overall HIV incidence of 0.64/100 PY [19]. WHO released guidelines for PrEP use for MSM in 2012 [20]. However, as of December 2017, no country in Asia has offered PrEP beyond the context of demonstration projects. PrEP can be obtained only in the context of research or through a commercial vendor in China.

The cost–benefit analysis of an intervention is highly relevant for policy implementation. Several modeling studies have addressed the excellent cost-effectiveness of ART [21, 22]. The HIV incidence among Chinese MSM of more than 3/100 PY is sufficient to implement PrEP based on WHO guidelines [3, 8], but the expense of PrEP likely prevents it from being a cost-effective option [23, 24]. The only cost-effectiveness study of PrEP in China to date concluded that the cost would need to be reduced by 50% to achieve cost-effectiveness [25]. However, several studies have shown the cost-effectiveness of ART provision to HIV-infected individuals in combination with PrEP for their sexual partners in high-prevalence settings [26–28]. As the health system in China begins to evaluate the feasibility of implementing PrEP in MSM through multicenter real-world studies [29], PrEP is likely to become an option for MSM in China. Modeling and cost-effectiveness analysis is needed urgently to understand the cost-effectiveness of different intervention strategies (including ART and PrEP) for MSM in China to provide guidance for policy decisions.

This prospective cohort study documented the effectiveness of early ART according to viral load (VL) suppression and changes in risk behaviors over 36 months post-infection. Based on these observed data, a mathematical model to calculate onward HIV transmission was created. Finally, cost-effectiveness analysis was conducted to estimate the cost, quality-adjusted life-years (QALYs), and cost-utility ratio (CUR) for six intervention scenarios that integrated ART with PrEP among MSM partners.

## Methods

### Ethical approval of the study protocol

The study protocol was approved by the Institutional Review Board of the First Affiliated Hospital of China Medical University ([2013]2011-36-2; Shenyang, China). All study participants provided written informed consent before initiation of any study-related collection of data or blood. This study was carried out in accordance with the relevant guidelines of China.

### Study setting

A prospective cohort study to identify and enroll participants with EHI was conducted in Shenyang, China. HIV-negative MSM were followed up every 2–3 months and were tested by pooled nucleic acid amplification testing of HIV-1. Between April 2009 and March 2016, 224 cases of EHI were identified. Details on this cohort have been published [30]. We enrolled 216 participants based on individuals being 18 years or older and having a diagnosis within 6 months of HIV infection. Participants were encouraged to initiate ART immediately. At each visit—3, 6, 12, 18, 24, and 36 months after HIV infection—participants received a physical examination, VL testing, and were asked questions about sexual activity during the past month.

For analyses, we divided participants into three groups according to the timing of ART initiation: “non-ART” for those who did not initiate ART between 0 months to 36 months post-infection, “standard-ART” for those who initiated ART 13 months to 36 months post-infection, and “early-ART” for those who received ART within 12 months post-infection.

### Data collection

We collected descriptive demographic data and the characteristics of sexual contacts at each visit. The number of sexual partners, the frequency of sexual activity, and the use of condoms with regular sex partners and casual sex partners were assessed through standardized questionnaires (Additional file 1) completed by participants in a private room. VL was measured by the COBAS® AmpliPrep/COBAS® TaqMan® HIV-1 test v2.0 (Roche Molecular Systems, Pleasanton, CA, USA) with analyses based on the lower limit of detection of 50 copies/mL. The estimated date of HIV infection was evaluated from the information provided by completed questionnaires and medical records. Statistical analyses were undertaken with SAS 9.2 (SAS, Cary, NC, USA). Social demographic and sexual behaviors data were presented as number and percentages for categorical variables, and continuous data were expressed as median (interquartile range [IQR]). The frequencies of categorical variables were compared using Chi-square test or Fisher’s exact test. Kruskal-Wallis test were used to compare means of 3 groups of variables not normally distributed. *P* < 0.05 was considered statistically significant.

### Model to calculate risk

Data on time-dependent VL and the frequency of transmission-related sexual behaviors were used to inform a simulative model. The latter was adjusted by the transmission probability per act associated with a VL setpoint, estimated HIV incidence of 5.61/100 PY [8], and an estimated HIV prevalence of 10% [31–34] among MSM in China. Then, this model was used to estimate the number of onward HIV transmissions per patient by 36 months post-infection.

Three main scenarios were modeled based on observed data, and were named non-ART, standard-ART, and early-ART, respectively. Another three hypothetical scenarios were modeled with the assumption of PrEP implementation among HIV-negative sexual partners: (i) non-ART plus partners’ PrEP (in which participants received medical care without ART and all of their sexual partners were assumed to take daily PrEP); (ii) standard-ART plus partners’ PrEP (in which participants received ART 13–36 months post-infection, and all sexual partners were assumed to take daily PrEP until their partners reached undetectable VL); (iii) early-ART plus partners’ PrEP (in which participants received ART within 12 months post-infection, and all sexual partners were assumed to take daily PrEP until their partners reached undetectable VL) (Additional file 3: Figure S1). PrEP was assumed to be 90% effective for preventing infection [14–17, 19].

Adaptation of an equation described previously was used to estimate the number of cases linked to an HIV-infected participant over a given duration [35–37]. Importantly, our model assumed that transmission risk behaviors remained constant throughout each follow-up interval across all scenarios [36].

$$ \lambda =n\left(1-P\right)\left\{1-{\left(1-{\beta}_1\right)}^{\frac{m}{n}}{\left(1-\left(1-\varepsilon \right){\beta}_1\right)}^{\frac{k}{n}}\right\} $$

where *n* is the average number of sexual partners for an EHI, *P* is the HIV prevalence among sexual partners of a person with EHI, which is estimated at 10% [31–34], so *n (1 − P)* is the number of sexual partners without HIV, *m* and *k* are the number of condom and condomless sexual acts, respectively, and *ε* is the protective efficiency of condom use (80%) [38].

Recently, Kroon et al. examined the relationship between VL and HIV transmission [36]. The transmissibility per average unprotected act per person was estimated to be:

$$ {\beta}_1={\gamma}^{{\mathit{\log}}_{10}\left(\frac{\nu_1}{\nu_0}\right)}{\beta}_0 $$

where *γ* is derived from a meta-analysis which found a 2.89-fold increase in infectiousness per tenfold increase in VL among MSM and male sexual partners [38]. The parameter *ν*_*1*_ is the current VL during any follow-up interval and *ν*_*0*_ is the VL setpoint among non-ART individuals in Chinese MSM (4.28 log_10_ copies/mL) [39], and *β*_*0*_ is calibrated to reflect the HIV incidence among MSM in China [8]. The calculated value of *β*_*0*_ was inferred to be 0.0038 (95% credible interval (CI): 0.0028–0.0052) (Additional file 1); this value is in reasonable agreement with an independent meta-analysis [38].

### Cost-effectiveness analysis

The economic-analysis outcomes were cost, QALYs, and CUR. The yearly cost of the non-ART scenario comprised regular medical care and management of opportunistic infections. The yearly cost of ART comprised assessment and initiation of ART, regular medical care after ART, ART drugs, management of opportunistic infections and side effects, and indirect medical costs. The yearly cost of PrEP comprised quarterly screening for HIV and sexually transmitted infections (STIs), treatment for STIs, PrEP drugs, regular medical care, and indirect medical costs (Additional file 2 and Additional file 3). All costs are reported in 2015 USD, and discounted at a 3% annual rate [40].

The QALYs included the reductions stemming from HIV diagnosis and from living with HIV. According to the *Sixth National Census Report* from 2010, Chinese men have an average lifespan of 72.4 years [41]. MSM were estimated to be HIV-positive at the age of 29.3 years in the present study. Considering the effects of ART adherence along with its side effects, life expectancy after initiation of ART has been estimated to be 30 years [42]. Based on the health utility value (Additional file 2: Table S4), the average individual loses an additional 10.0 QALYs due to opportunistic infections while on ART over this 30-year horizon [43]. In summary, each person who is diagnosed with HIV loses 23.1 QALYs over his lifetime, and loses 2.3 QALYs by 36 months post-infection. CUR estimations were calculated through dividing costs by QALYs, which were calculated by the number of HIV infections averted in each scenario. Based on the WHO principle of cost benefit [44, 45], a strategy was “very cost-effective” if CUR was less than the average of China’s per capita gross domestic product from 2009 to 2015 ($6,172) [46], and was “cost-effective” if CUR was less than three-times China’s per capita gross domestic product ($18,515).

### Sensitivity analyses

Sensitivity analyses were used to evaluate the impact of critical parameters on cost-effectiveness: HIV prevalence, HIV incidence, PrEP efficacy, PrEP drug cost per day, ART drug cost per day, and life expectancy after ART initiation.

## Results

### Sociodemographic characteristics

Baseline characteristics are shown in Table 1. According to the timing of ART initiation, 92, 28, and 96 participants were classified as non-ART, standard-ART, and early-ART, and 65, 46, and 86% of each group was retained at the visit 36 months post-infection, respectively. The average estimated duration of HIV infection at diagnosis was 49 (interquartile range: 31–76) days. The proportion of married individuals and people older than 35 years was higher in the standard-ART group, and more patients in the early-ART group than in the other groups found male sex partners through the Internet (*P* < 0.05 for all).

**Table 1:** Baseline sociodemographic characteristics of EHI

Variable	Total	Non-ART	Standard-ART	Early-ART	*P* value
Total	216(100.0)	92(100.0)	28(100.0)	96(100.0)	NA
age (years)					<0.01^a^
≤24	75(34.7)	35(38.0)	3(10.7)	37(38.5)	
25–34	86(39.8)	36(39.1)	8(28.6)	42(43.8)	
≥35	55(25.5)	21(22.8)	17(60.7)	17(17.7)	
Median (IQR)	26.7 (22.7–34.2)	26.0 (22.0–32.6)	38.8 (26.2–44.1)	26.1 (22.7–31.1)	
Residence permit					0.22 ^a^
Shenyang	81(37.5)	33(35.9)	8(28.6)	40(41.7)	
Other city of Liaoning province	94(43.5)	37(40.2)	17(60.7)	40(41.7)	
Other province	41(19.0)	22(23.9)	3(10.7)	16(16.7)	
Ethnicity					0.05 ^a^
Han	185(85.6)	83(90.2)	26(92.9)	76(79.2)	
Others	31(14.4)	9(9.8)	2(7.1)	20(20.8)	
Marital status					0.01^b^
Single	158(73.1)	71(77.2)	13(46.4)	74(77.1)	
Married	34(15.7)	9(9.8)	11(39.3)	14(14.6)	
Divorced or widowed or unknown	24(11.1)	12(13.0)	4(14.3)	8(8.3)	
Education (years)					0.11 ^a^
≤12	109(50.5)	51(55.4)	17(60.7)	41(42.7)	
>12	107(49.5)	41(44.6)	11(39.3)	55(57.3)	
Occupation					0.28 ^a^
Employed	85(39.4)	39(42.4)	9(32.1)	37(38.5)	
Unemployed	48(22.2)	20(21.7)	5(17.9)	23(24.0)	
Student	28(13.0)	8(8.7)	3(10.7)	17(17.7)	
Self-employed	55(25.5)	25(27.2)	11(39.3)	19(19.8)	
Monthly income ($)					0.15 ^a^
≤540	183(84.7)	78(84.8)	27(96.4)	78(81.3)	
>540	33(15.3)	14(15.2)	1(3.6)	18(18.8)	
Main location for seeking same-sex male partners					<0.01 ^b^
Internet	155(71.8)	60(65.2)	14(50.0)	81(84.4)	
Park or public bath	26(12.0)	11(12.0)	11(39.3)	4(4.2)	
Others	35(16.2)	21(22.8)	3(10.7)	11(11.5)	
Duration of infection at diagnosis					0.03^c^
Median (IQR)(days)	49(31–76)	49(30–79)	68(48–105)	43(31–69)	
Number of male sexual partners					0.09^c^
Median (IQR)	2(1–3)	2(1–3)	3(2–4)	2(1–2)	
Number of sexual behaviors with male partners					0.27^c^
Median (IQR)	3(1–5)	3(1–6)	4(2–6)	2(1–5)	

^a^: chi-square test; ^b^: Fisher exacted test; ^c^: Kruskal-Wallis test; EHI: early HIV infection; ART: antiretroviral therapy; NA: not applicable, IQR: interquartile range; $: U.S. dollars

### Trends in change of UAI and VL

**Fig. 1:**
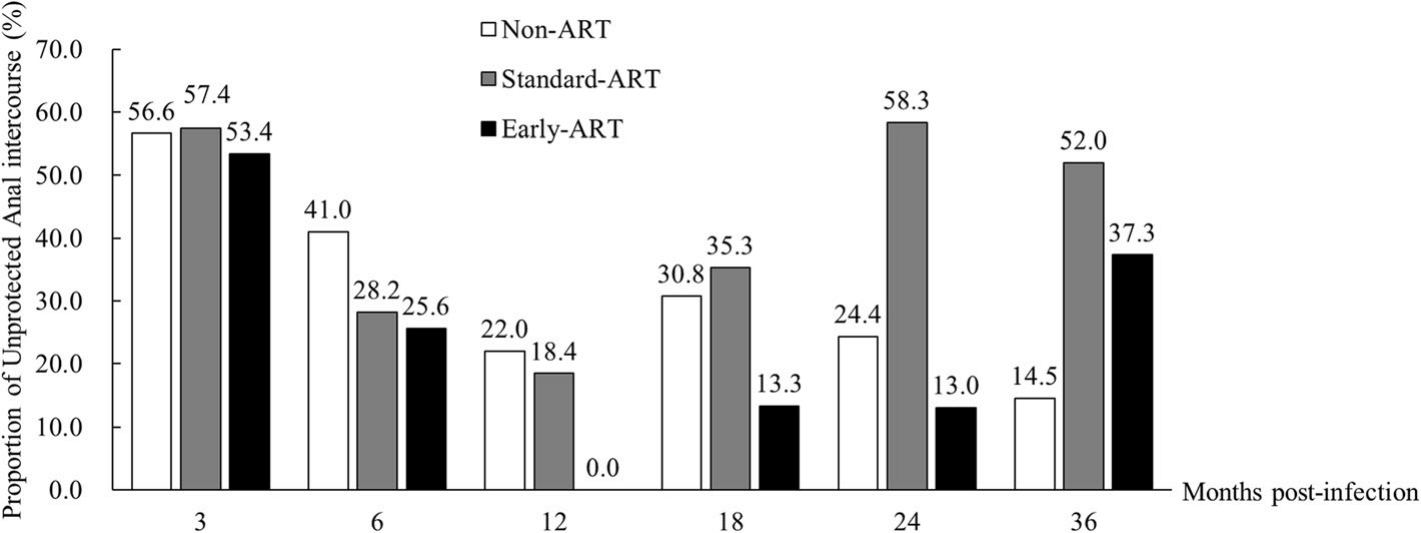
percentage of self-reported unprotected anal intercourse

In the 36 months after HIV diagnosis, the percentage of participants self-reporting UAI decreased (Fig. 1). In the early-ART group, the average number of anal-sex acts per person per month at baseline (i.e., the month before diagnosis), 12 months, and 36 months was 3.2, 1.3, and 3.1 (data not shown), whereas the proportion of UAI was 53.4, 0.0, and 37.3%, respectively. In the non-ART group, the average number of sexual behaviors per person per month at baseline, 12 months, and 36 months was 4.6, 3.1, and 6.9 (data not shown), whereas the proportion of UAI was 56.6, 22.0, and 14.5%, respectively.

**Fig. 2:**
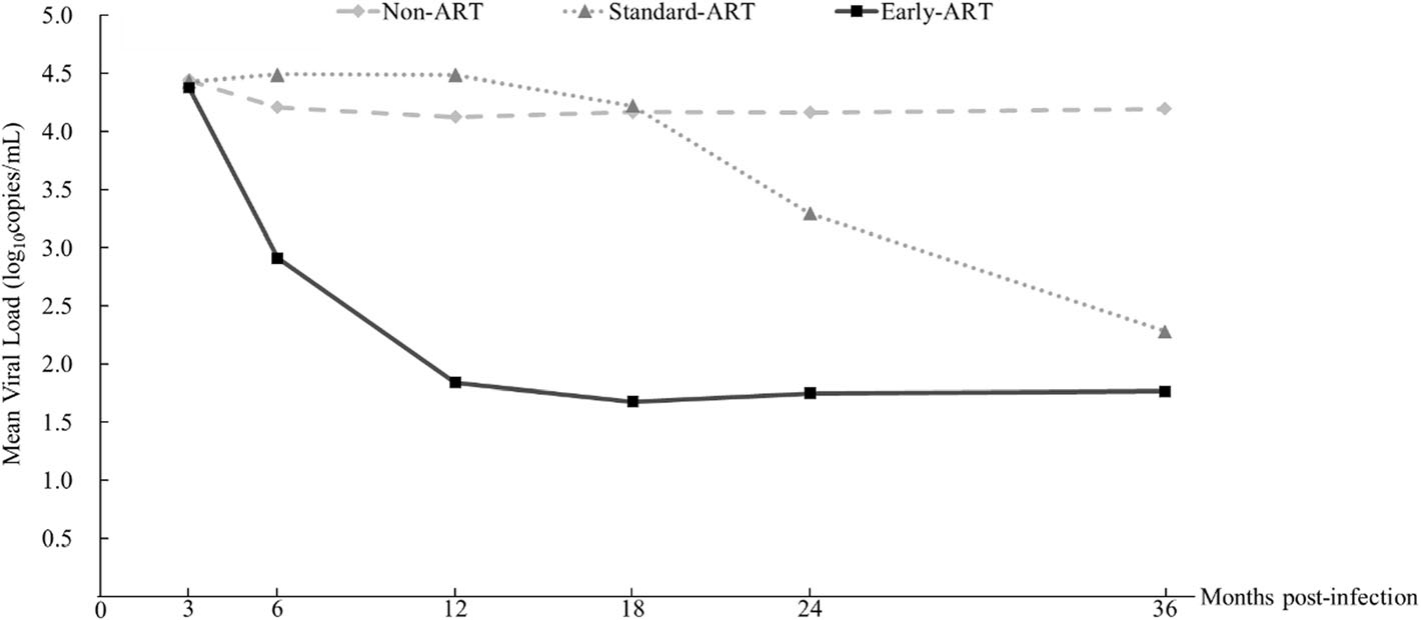
Viral load change trend over 36 months post-infection

The mean decline in VL is shown in Fig. 2. Individuals in the non-ART group stabilized at 4.2 log_10_ copies/mL by 6 months post-infection. ART was initiated, on average, 3 months post-infection in the early-ART group, and VL decreased sharply, on average, to 50 copies/mL after 12 months. Meanwhile, the timing of ART initiation in the standard-ART group was 23 months post-infection; VL was high before ART initiation and decreased rapidly, on average, to 200 copies/mL by 36 months post-infection.

### Estimated HIV transmission per 100 cases over 36 months post-infection

Based on VL and UAI, the estimated number of HIV transmissions per 100 cases per month was illustrated in six scenarios (Table 2). Based on observed data, the number of HIV transmissions per 100 cases in the non-ART, standard-ART, and early-ART scenarios was 2.79, 1.85, 1.29 at 3 months, 1.26, 1.81, 0.03 at 12 months, and 0.65, 0.44, 0.26 at 36 months, respectively. Based on observed and hypothetical data, the number of HIV transmissions per 100 cases in the non-ART plus partners’ PrEP, standard-ART plus partners’ PrEP, and early-ART plus partners’ PrEP scenarios was 0.28, 0.19, 0.13 at 3 months, 0.13, 0.18, 0.01 at 12 months, and 0.06, 0.04, 0.03 at 36 months, respectively.

**Table 2:** Estimated HIV transmissions per 100 cases at 36 months post-infection

Scenario	Month 3	Month 6	Month 12	Month 18	Month 24	Month 36	Sum over 36 months post-infection
Scenarios based on observed data							
Non-ART	2.79(2.05–3.85)	1.41(1.04–1.95)	1.26(0.93–1.74)	1.60(1.18–2.21)	0.71(0.52–0.98)	0.65(0.48–0.90)	41.83(30.75–57.69)
Standard-ART	1.85(1.36–2.56)	1.77(1.30–2.45)	1.81(1.33–2.50)	0.47(0.34–0.65)	0.44(0.32–0.61)	0.44(0.32–0.61)	32.51(23.89–44.85)
Early-ART	1.29(0.95–1.78)	0.07(0.05–0.09)	0.03(0.02–0.04)	0.04(0.03–0.06)	0.05(0.04–0.07)	0.26(0.19–0.36)	7.95(5.85–10.95)
Scenarios based on observed and hypothetical data							
Non-ART plus partners’ PrEP	0.28(0.21–0.38)	0.14(0.10–0.19)	0.13(0.09–0.17)	0.16(0.12–0.22)	0.07(0.05–0.10)	0.06(0.05–0.09)	4.18(3.07–5.77)
Standard-ART plus partners’ PrEP	0.19(0.14–0.26)	0.18(0.13–0.24)	0.18(0.13–0.25)	0.05(0.03–0.06)	0.04(0.03–0.06)	0.04(0.03–0.06)	3.25(2.39–4.48)
Early-ART plus partners’ PrEP	0.13(0.10–0.18)	0.01(0.01–0.01)	0.01(0.01–0.01)	0.01(0.01–0.01)	0.01(0.01–0.01)	0.03(0.02–0.04)	0.79(0.58–1.09)

*ART* antiretroviral therapy, *PrEP* pre-exposure prophylaxis

**Fig. 3:**
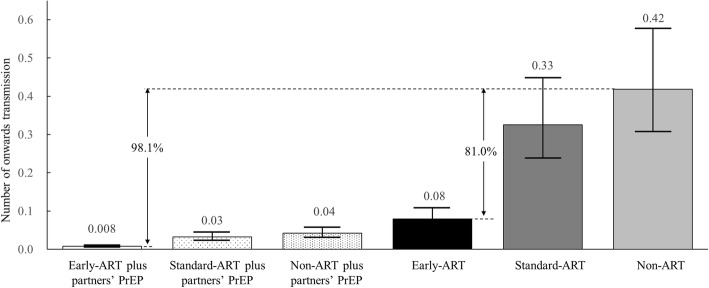
Estimated HIV transmissions per 100 cases in 6 scenarios over 36 months post-infection

Next, the total number of new HIV cases 36 months post-infection was evaluated in the six scenarios we tested. The estimated total was 41.83 (95%CI: 30.75 to 57.69) in the non-ART, 32.51 (23.89 to 44.85) in standard-ART and 7.95 (5.85 to 10.95) in early-ART scenarios. These results translated into an estimated 81.0% reduction in HIV transmission due to early treatment. The hypothetical scenario of early-ART plus partners’ PrEP yielded an estimated 0.79 (95%CI: 0.58 to 1.09) HIV new cases, which was an estimated 98.1% reduction compared with the non-ART scenario (Table 2 and Fig. 3).

### Cost-effectiveness over 36 months post-infection

The cost was $3,540 for the non-ART, $6,063 for standard-ART, and $10,024 for early-ART scenarios. The cost was $33,973 for standard-ART plus partners’ PrEP, and $15,872 for early-ART plus partners’ PrEP. The non-ART plus partners’ PrEP scenario incurred the highest cost ($40,974).

Compared with the non-ART scenario, the scenarios of standard-ART, early-ART, non-ART plus partners’ PrEP, standard-ART plus partners’ PrEP, and early-ART plus partners’ PrEP had a CUR of $28,272, $12,864, $47,321, $38,287, and $16,817 per QALY, respectively. Early-ART and early-ART plus partners’ PrEP were cost-effective (Table 3).

**Table 3:** Cost-utility ratio per case in 6 scenarios over 36 months post-infection

Scenario	Cost ($)	Averted HIV infection	QALY	CUR
Scenarios based on observed data				
Non-ART	3,540	NA	NA	NA
Standard-ART	6,063	0.09	0.21	28,272
Early-ART	10,024	0.34	0.78	12,864
Scenarios based on observed and hypothetical data				
Non-ART plus partners’ PrEP	40,974	0.38	0.87	47,321
Standard-ART plus partners’ PrEP	33,973	0.39	0.89	38,287
Early-ART plus partners’ PrEP	15,872	0.41	0.94	16,817

*$* U.S. dollars, *QALY* quality-adjusted life-years, *CUR* cost-utility ratio, *ART* antiretroviral therapy, *NA* not applicable, *PrEP* pre-exposure prophylaxis

### Sensitivity analyses

**Fig. 4:**
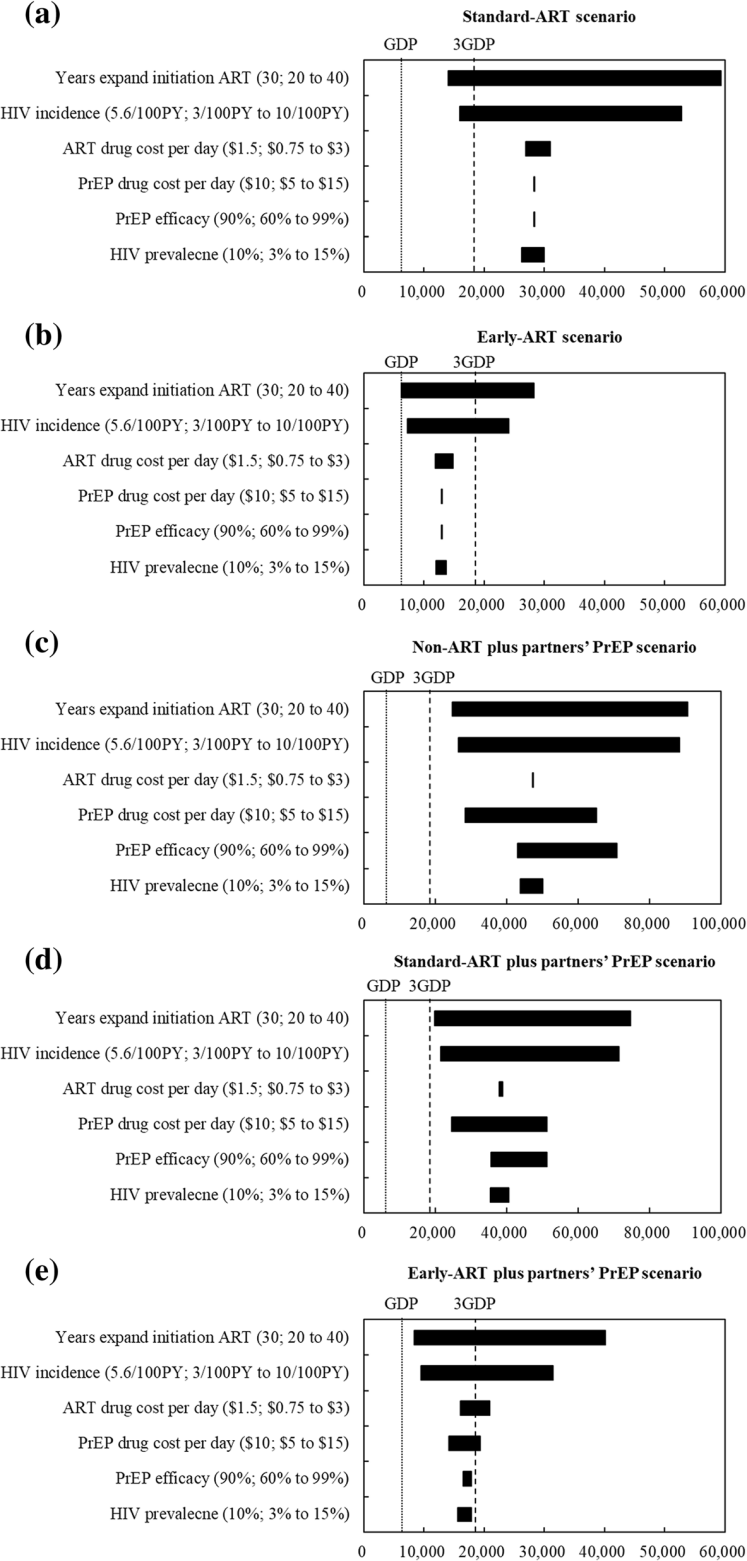
Tornado diagrams of one-way sensitivity analysis of the CUR compared to the status scenario

Figure 4 presents the one-way sensitivity analysis of the CUR for different scenarios compared with the non-ART scenario. Our overall finding was that early-ART and early-ART plus partners’ PrEP remained cost-effective within all uncertainty in the input parameters. Importantly, parameters such as ART drug cost per day, PrEP drug cost per day, and HIV prevalence, had a minimal impact on the CUR.

## Discussion

The results of the present study provide evidence that early initiation of ART reduces HIV transmission substantially among MSM. Early ART with reduction in VL along with changes in sexual behaviors was estimated to reduce HIV transmission by 81.0% in the initial 36 months of infection. PrEP provision to sexual partners of participants who belonged to the early-ART group with unsuppressed VL could nearly eradicate HIV-1 transmission. Economic analyses suggested that early-ART and an integrated package of early-ART plus partners’ PrEP were cost-saving and led to health benefits up to 36 months post-infection.

Treatment as prevention for MSM is indeed effective. The Opposites Attract study detected no phylogenetically linked HIV transmission events in 16,800 acts of UAI [2]. Those results confirmed the PARTNER study, which noted no linked HIV transmission events among same-sex male couples [1]. However, more information is needed to understand the effectiveness of treatment as prevention during the EHI stage [36].

Participants in the present study were divided into early-ART, standard-ART, and non-ART groups according to the most current (from 2009 to 2016) ART-initiation standards in China. All HIV-infected individuals received immediate ART after July 2016 based on updated Chinese clinical guidance [4]. Therefore, this cohort represents a valuable population in which to assess the benefits of early ART initiation in the real world. Our study offers the first evaluation of the possible results of early ART initiation based on observed effects among MSM in China.

Compared with the non-ART group, the early-ART group showed greatly reduced HIV transmission over the first 36 months of infection. We found that early initiation of ART could reduce 81.0% of HIV transmission. This reduction was lower than that found in the RV254/SEARCH 010 Study Group, which showed that early ART for acute HIV infection (AHI) could reduce transmission by 88.9% [36]. Possible explanations for this difference were: that participants in our study had EHI and started ART later (on average, 3 months in our study compared with, on average, 0.5 months in RV254); the type of study design (real-world data in our study compared with a randomized controlled trial in RV254).

EHI is a very important stage for HIV transmission [9, 10, 47]. Our results showed that early ART could reduce the risk of transmission significantly, and that integration of early-ART plus partners’ PrEP could stop HIV transmission. Hence, the increase in HIV testing and early linkage to ART is an important strategy in preventing HIV among MSM.

Although identifying individuals with AHI or EHI is challenging, cost-effectiveness may be achieved by adding pooled nucleic acid amplification testing to diagnostic algorithms for MSM [30, 48]. An increase in self-reported UAI in the early-ART group was observed over time. This was accompanied by the number of HIV transmissions at 36 months post-infection being higher than those at 6, 12, and 24 months. This phenomenon may not be a form of risk compensation: if VL is suppressed, UAI between a serodiscordant same-sex relationship is less risky (undetectable = untransmittable) [49]. HIV may be untransmittable, but sexually transmitted diseases (STIs) should be monitored in this population, particularly those who are asymptomatic.

In addition, the early-ART and early-ART plus partners’ PrEP scenarios were cost-effective when the current prices of ART and PrEP in China were taken into account. The non-ART plus Partners’ PrEP group had the highest CUR, which may have been caused by the relatively high price of PrEP. Zhang and colleagues [25] described a compartmental model for evaluating the cost-effectiveness of PrEP in Chinese MSM. They found that it could achieve cost-effectiveness unless the cost of PrEP was reduced by 50%. More importantly, the early-ART plus partners’ PrEP group had a 98.1% decrease in HIV transmission and was cost-effective. Most individuals achieve viral suppression after ART, so their sexual partners are unlikely to be at risk of HIV infection and do not need PrEP. However, in the first 6 months after ART initiation or if the virus rebounds during ART, sexual partners are recommended to use PrEP to avoid HIV infection. Studies in Africa have shown that implementation of PrEP until sustained ART use among HIV-1-serodiscordant couples can almost eliminate HIV-1 transmission [27], and are cost-effective [26, 44]. Shen and colleagues [50] developed a mathematical model and found that high PrEP coverage with earlier ART initiation was cost-effective for MSM in San Francisco, USA. PrEP guidelines have not been introduced to MSM in China. Given the current high cost of tenofovir/emtricitabine in China and resource constraints, our analysis suggests that prioritizing early ART for HIV-positive individuals and PrEP for their HIV-negative sexual partners until they have achieved viral suppression would control the spread of HIV among Chinese MSM efficiently while saving costs. The results of our mathematical model clearly indicate the utility of the partners’ PrEP approach, but implementation of such a strategy will have significant challenges, particularly in an environment of high stigmatization of HIV [51] and MSM [52]. Formative research to consider the feasibility of partner notification with immediate offer of PrEP is needed if such a strategy is to be considered.

Our study had five main limitations. First, as with all mathematical models, the risk-equation model was a simplification of reality. Other factors, such as STI co-infection, were not considered, which increased uncertainty. The 95% CI accommodated such potential variations in the main assumptions. Second, the estimated number of transmissions averted was determined by the relationship between VL and HIV transmission probabilities. This relationship was adjusted by the VL setpoint for MSM in China and the magnitude of transmission probability from local epidemiology data. Therefore, one must be cautious when extrapolating conclusions. Third, self-reporting was employed to determine transmission-related sexual behaviors, which were subject to a bias in social desirability. Standardized questionnaires administered at each visit minimized these issues as much as possible. Fourth, the effect of biological intervention was evaluated for only 36 months after HIV infection. Future studies should explore the long-term benefits of early ART initiation and long-term cost-effectiveness of early ART combined with PrEP implementation. Finally, our analysis considered only the cost-effectiveness of daily oral PrEP; as more evidence accrues on the efficacy of event-driven PrEP, additional scenarios could be modeled to evaluate the cost effectiveness if event-driven PrEP is recommended.

## Conclusions

An integrated approach with early ART and sexual partners’ PrEP could almost stop HIV transmission and result in cost savings over 36 months post-infection. Therefore, this study offers evidence of a combination strategy that has the potential to result in large decreases in HIV transmission and, thus, contribute to controlling the HIV epidemic among MSM in China.

## Additional files

Additional file 1: Estimation for transmission probability *β*_0_. (DOCX 21 kb)

Additional file 2: Estimated cost of no-ART, ART and PrEP per person per year. (DOCX 25 kb)

Additional file 3: Estimated cost to implement 6 scenarios over 36 months post-infection. (DOCX 107 kb)

## Data Availability

An anonymized dataset and all statistical codes are available upon reasonable request from the corresponding author.

## Abbreviations

ART: Antiretroviral therapy
CI: Credible interval
CUR: Cost-utility ratio
EHI: Early HIV infection
HIV: Human immunodeficiency virus
MSM: Men who have sex with men
PrEP: Pre-exposure prophylaxis
QALYs: Quality-adjusted life-years
STIs: Sexually transmitted infections
UAI: Unprotected anal intercourse
VL: Viral load
