# Supplementary material for: Efficacy and cost-effectiveness of early antiretroviral therapy and partners’ pre-exposure prophylaxis among men who have sex with men in Shenyang, China: a prospective cohort and costing study
Source: BMC Infect Dis. 2019 Jul 25;19:663. doi: 10.1186/s12879-019-4275-x (PMC6659226; doi:10.1186/s12879-019-4275-x)
Supplement: Supplementary file 1 — Estimation for transmission probability β0. (DOCX 21 kb) [file 12879_2019_4275_MOESM1_ESM.docx]

**Additional file 1****: Estimation for transmission probability *β₀***

The average frequency of sexual encounters is approximately once per week for Chinese adults [1]. The proportion of unprotected sexual behaviors of individuals unaware of their HIV serostatus has been reported to be 3.5-times higher than in those who are aware of their HIV serostatus [2]. Therefore, the frequency of sex is reduced to approximately 1/3.5 of the frequency pre-infection. Therefore, the number of sexual behaviors per person per year is 14.86 among those aware of their HIV status. The current HIV incidence among MSM in China is 5.61/100 person-years [3], so the risk of HIV transmission is 0.0561 per person per year. Therefore, the risk of HIV transmission per sex act in this population is 0.003776 (95% confidence interval: 0.002774–0.005212). This result is in reasonable agreement with independent pooled estimates of transmission risk calculated by separate meta-analyses for anal intercourse [4].

**Reference**

[1] Pan S, Wang A. Sexual behavior and relation in contemporary China. Beijing, 2004. Chinese.

[2] Marks G, Crepaz N, Janssen RS. Estimating sexual transmission of HIV from persons aware and unaware that they are infected with the virus in the USA. AIDS. 2006;20(10):1447-50.

[3] Zhang W, Xu JJ, Zou H, Zhang J, Wang N, Shang H. HIV incidence and associated risk factors in men who have sex with men in mainland china: An updated systematic review and meta-analysis. Sexual health. 2016;13(4):373-82.

[4] Patel P, Borkowf CB, Brooks JT, Lasry A, Lansky A, Mermin J. Estimating per-act HIV transmission risk: A systematic review. AIDS. 2014;28(10):1509-19.

**Questionnaire of Early HIV infection**

*Please draw a mark in the box before the correct option.*

1. Patient ID:__________

2. Patient group: 1) early-ART 2) standard-ART 3) non-ART

3. Survey time-point:

1) 3 month post-infection 2) 6 month post-infection 3) 12 month post-infection

4) 18 month post-infection 5) 24 month post-infection 6) 36 month post-infection

**Part 1: background information**

1. Place of residence: _____ province ____ city _____ county/district

2. Date of birth: Year____ Month______ Day______

3. Ethnicity:

1) Han 2) Others: _______

4. Current marital status:

1) Single 2) Married 3) Separated from wife 4) Cohabitation with girlfriend

5) Cohabitation with male partner 6) Divorce 7) Widowed

5. Education:

1) Not attending school 2) Elementary school 3) Junior high school

4) High school 5) University/college and above

6. Current occupation:

1) Freelancers 2) Private owners/individual merchants 3) Farmers 4) Factory workers 5) Students 6) Unemployed 7) Company Staff 8) Others: ________

7. Current average monthly income: ______Yuan (without income, fill in “0” yuan)

8. How do you behave when you have sex with other men?

1) Always bottom ("0") 2) Always top ("1")

3) both of top and bottom 4) Other ________

9. Main location for seeking homosexual partners:

1) Internet 2) Hotel/hotel 3) Nightclubs / dance halls 4) Public baths

5) Public toilets 6) Park 7) Gay Club 8) Other _______________

10. Smoking: 1) No (Jump to 12) 2) Yes

If smoke, how many years have you been smoking? ______Years

How many cigarettes have you been smoking per day? _____ cigarettes per day

11. Drinking: 1) No (Jump to part 2) 2) Yes

If drink, how many years have you been drinking? ______Years

How many times have you been drinking per week? _____ times per week

**Part 2: Sexual behavior**

Please recall the number of male sexual partners and condom usage information in the past one month

| Sexual partners type | Number of partners | Number of HIV^+^ partners | Anal sex as top | | Anal sex as bottom | |
| --- | --- | --- | --- | --- | --- | --- |
|  |  |  | Number of sexual intercourse | Number of condom usage^*^ | Number of sexual intercourse | Number of condom usage^*^ |
| Primary sexual partners |  |  |  |  |  |  |
| Casual sexual partners |  |  |  |  |  |  |
| Commercial sexual partners -purchaser |  |  |  |  |  |  |
| Commercial sexual partners -seller |  |  |  |  |  |  |
| Total |  |  |  |  |  |  |

^*^: During the sexual intercourse, participants did not use condom throughout or condom broken will be considered as unused condoms.

Investigator:_____________________ Date of investigation:__________________
