# Supplementary material for: Efficacy and cost-effectiveness of early antiretroviral therapy and partners’ pre-exposure prophylaxis among men who have sex with men in Shenyang, China: a prospective cohort and costing study
Source: BMC Infect Dis. 2019 Jul 25;19:663. doi: 10.1186/s12879-019-4275-x (PMC6659226; doi:10.1186/s12879-019-4275-x)
Supplement: Supplementary file 2 — Estimated cost of no-ART, ART and PrEP per person per year. (DOCX 25 kb) [file 12879_2019_4275_MOESM2_ESM.docx]

**Additional File 2:** **Estimated cost of no-ART, ART, and PrEP per person per year**

Table S1 Estimated cost of non-ART per year

| Category | | Price per unit ($) | | Frequency per year | Total($) |
| --- | --- | --- | --- | --- | --- |
| Regular medical care | |  | |  |  |
| CD4 testing | | 36(32-40) | | 2 | 72(65-80) |
| VL testing | | 208(200-215) | | 1 | 208(200-215) |
| Follow-up | | 18(13-23) | | 2 | 36(26-45) |
| Opportunistic infections management | | | |  |  |
| Cost of hospital care | | 829(647-1005) | | 1 | 829(647-1005) |
| Cost of outpatient care | | 35(30-41) | | 1 | 35(30-41) |
| Sum | NA | | NA | | 1180(968-1387) |

ART: antiretroviral therapy; $: U.S. dollars; VL: viral load; NA: not applicable.

Table S2 Estimated cost of ART within 36 months post-infection

| Category | Price per unit ($) | Frequency per year | 12 months post-infection  ($) | 13-36 months post-infection  ($) |
| --- | --- | --- | --- | --- |
| Assessment and initiation of ART | 67(54-81) | 1 | 67(54-81) | NA |
| Regular medical care after ART |  |  |  |  |
| CD4 testing | 36(32-40) | 2 | 72(65-80) | 145(129-160) |
| VL testing | 208(200-215) | 1 | 208(200-215) | 415(400-431) |
| Follow-up fee | 18(13-23) | 4^a^ | 71(52-90) | 71(52-90) |
| ART drugs |  |  |  |  |
| First line drugs | 505(457-554) | 0.95^b^ | 480(434-526) | 960(868-1052) |
| Second line drugs | 1098(923-1272) | 0.05^b^ | 55(46-64) | 110(92-127) |
| Management of opportunistic infections and side effects |  |  |  |  |
| Cost of hospital care | 1093(864-1323) | 1 | 1093(864-1323) | 2187(1729-2645) |
| Cost of outpatient care | 448(402-494) | 1 | 448(402-494) | 896(803-989) |
| Indirect medical costs |  |  |  |  |
| Transport | 143 | 1 | 143 | 286 |
| Care services | 152 | 1 | 152 | 304 |
| Nutritional costs | 110 | 1 | 110 | 219 |
| Working hours lost | 645 | 1 | 645 | 1290 |
| Staff fee for ART administration | 37 | 1 | 37 | 74 |
| Staff fee for lab testing | 15 | 1 | 15 | 31 |
| Staff fee for HIV positive patients' administration | 15 | 1 | 15 | 31 |
| Sum | NA | NA | 3612  (3233-3991) | 7019  (6308-7730) |

^a^: 4 times in the first year of ART and 2 times after first year of ART; ^b^: according to the proportion of primary drug resistance, 95% patients were provided with first line prescription and 5% were second line prescription; ART: antiretroviral therapy; $: U.S. dollars; VL: viral load; NA: not applicable.

Table S3 Estimated cost of PrEP per year

| Category | Price per unit ($) | Frequency per year | Total ($) |
| --- | --- | --- | --- |
| HIV screening | 12(9-14) | 4 | 46(37-55) |
| STIs testing and treatment |  |  |  |
| STIs test (including sypgilis, gonorrhea and chlamydia) ^a^ | 38(31-46) | 4 | 154(123-185) |
| Syphilis treated | 38(31-46) | 0.1^b^ | 4(3-5) |
| Gonorrhea treated | 77(62-92) | 0.18^b^ | 14(11-17) |
| Chlamydia treated | 77(62-92) | 0.13^b^ | 10(8-12) |
| PrEP drugs ^d^ | 10 | 365 | 3706 |
| Regularly medical care |  |  |  |
| Liver and kidney function | 11(9-14) | 4 | 45(35-55) |
| Blood routine test | 3(3-3) | 4 | 12(11-12) |
| Urine routine test | 4(4-4) | 4 | 16(15-17) |
| Bone density examination | 31(26-35) | 1 | 31(26-35) |
| Hepatitis | 15(11-20) | 1 | 15(11-20) |
| Indirect medical costs |  |  |  |
| Transport | 8 | 4 | 31 |
| Working hours lost ^c^ | 31 | 4 | 123 |
| Staff fee for PrEP drugs administration | 37 | 1 | 37 |
| Staff fee for lab testing | 31 | 1 | 31 |
| Staff fee for administration of PrEP patients | 15 | 1 | 15 |
| Sum | NA | NA | 4290(4224-4356) |

^a^: According to the WHO PrEP guideline, syphilis, gonorrhea and chlamydia should be tested for every quarter. ^b^: The current syphilis incidence is about 10% (Int J STD AIDS. 2017;28(2):170-178.), the current gonorrhea and chlamydia prevalence is about 18% and 13%, respectively (Int J STD AIDS. 2017;28(11):1115-1123.); ^c^: It takes 4 working days to visit the clinic, buy drugs and receive related tests every year. The average salary of Shenyang residents in 2015 (4361 RMB/month) is about 200 RMB per day; ^d^: The current price of Truvada is 1980 RMB per bottle in China, with a price of 66 RMB per pill; PrEP: pre-exposure prophylaxis; $: U.S. dollars; STIs: sexually transmitted infections; NA: not applicable.

Table S4 Health Utility Value in different disease stage^*^

| ART | CD4 count (cells/mm^3^) | Health Utility Value |
| --- | --- | --- |
| No | >350 | 0.74 |
| No | 201 to 350 | 0.73 |
| No | ≤200 | 0.72 |
| Yes | >350 | 0.69 |
| Yes | 201 to 350 | 0.66 |
| Yes | ≤200 | 0.65 |
| NA | Dead | 0 |

^*^: According to the number of CD4 cells before and after antiretroviral therapy (ART), the Markov model was set as seven different disease stages: CD4 cells ≤200 cells/mm3, 201 to 350 cells/mm3, >350 cells/mm3, ART and CD4 cells ≤200 cells/mm3, ART and CD4 cells 201 to 350 cells/mm3, ART and CD4 cells >350 cells/mm3 and death. ART: antiretroviral therapy, NA: not applicable.
