# Supplementary material for: Efficacy and cost-effectiveness of early antiretroviral therapy and partners’ pre-exposure prophylaxis among men who have sex with men in Shenyang, China: a prospective cohort and costing study
Source: BMC Infect Dis. 2019 Jul 25;19:663. doi: 10.1186/s12879-019-4275-x (PMC6659226; doi:10.1186/s12879-019-4275-x)
Supplement: Supplementary file 3 — Estimated cost to implement 6 scenarios over 36 months post-infection. (DOCX 107 kb) [file 12879_2019_4275_MOESM3_ESM.docx]

**Additional file 3: Estimated cost to implement 6 scenarios over 36 months post-infection**


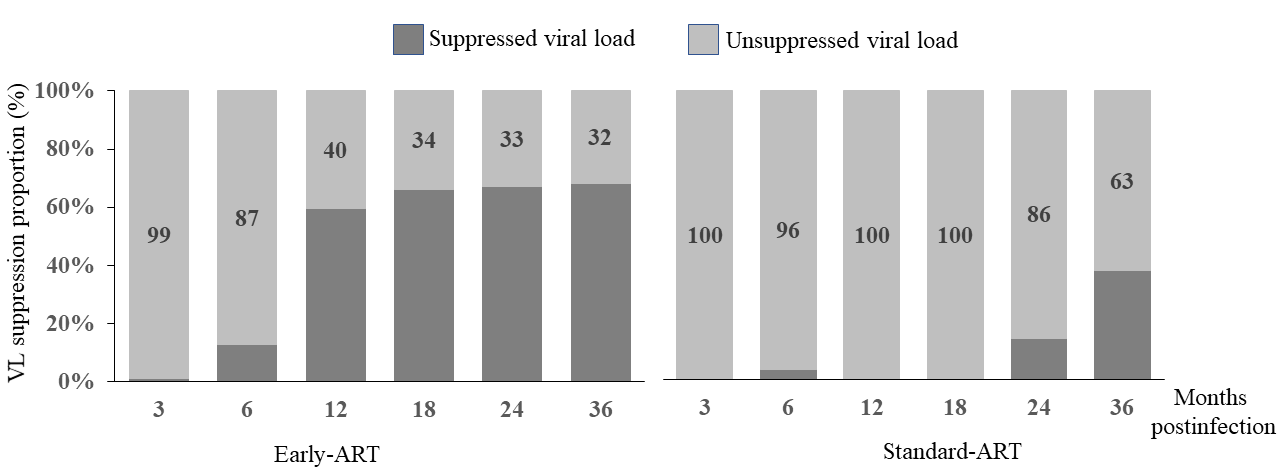


Figure S1 Proportion of viral load suppressed over 36 months post-infection in early-ART and standard-ART scenarios

Table S5 Estimated cost of non-ART scenario over 36 months post-infection

| Month^*^ | Non-ART cost per month ($) | | Cost within duration ($) |
| --- | --- | --- | --- |
| 3 | 98(81-116) | | 295(242-347) |
| 6 | 98(81-116) | | 295(242-347) |
| 12 | 98(81-116) | | 590(484-694) |
| 18 | 98(81-116) | | 590(484-694) |
| 24 | 98(81-116) | | 590(484-694) |
| 36 | 98(81-116) | | 1180(967-1387) |
| Sum | | NA | 3540(2902-4162) |

^*^: No patients received ART for over 36 months post-infection; ART: antiretroviral therapy; $: U.S. dollars; NA: not applicable.

Table S6 Estimated cost of standard-ART scenario over 36 months post-infection

| Month^*^ | Non-ART cost per month ($) | ART cost per month ($) | Cost within duration ($) |
| --- | --- | --- | --- |
| 3 | 98(81-116) | NA | 295(242-347) |
| 6 | 98(81-116) | NA | 295(242-347) |
| 12 | 98(81-116) | NA | 590(484-694) |
| 18 | 98(81-116) | NA | 590(484-694) |
| 23 | 98(81-116) | NA | 492(403-578) |
| 24 | NA | 292(263-322) | 292(263-322) |
| 36 | NA | 292(263-322) | 3510(3154-3865) |
| Sum | NA | NA | 6063(5271-6846) |

^*^: According the current ART guideline during 2009-2016, participants’ average timing of ART initiation was 23 months after HIV infection; ART: antiretroviral therapy; $: U.S. dollars; NA: not applicable.

Table S7 Estimated cost of early-ART scenario over 36 months post-infection

| Month^*^ | Non-ART cost  per month ($) | ART cost  per month ($) | Cost  within duration ($) |
| --- | --- | --- | --- |
| 3 | 98(81-116) | NA | 295(242-347) |
| 6 | NA | 301(269-333) | 903(808-998) |
| 12 | NA | 301(269-333) | 1806(1616-1996) |
| 18 | NA | 292(263-322) | 1755(1577-1933) |
| 24 | NA | 292(263-322) | 1755(1577-1933) |
| 36 | NA | 292(263-322) | 3510(3153-3866) |
| Sum | NA | NA | 10024(8973-11072) |

^*^: All patients received ART immediately after diagnosis. The average timing of ART initiation was 3 months after HIV infection; ART: antiretroviral therapy; $: U.S. dollars; NA: not applicable.

Table S8 Estimated cost of non-ART plus partners’ PrEP scenarios over 36 months post-infection

| Month^*^ | Number of sexual partners (mean) | Adjusted negative sexual partners^#^ | Non-ART cost per month ($) | Proportion unsuppressed (%) | PrEP cost per month ($) | Non-ART cost within duration ($) | PrEP cost within duration ($) | Cost within duration ($) |
| --- | --- | --- | --- | --- | --- | --- | --- | --- |
| 3 | 2.9 | 2.8 | 98  (81-116) | 100.0 | 358  (352-363) | 295  (242-347) | 3003  (2957-3050) | 3298  (3199-3397) |
| 6 | 2.7 | 2.5 | 98  (81-116) | 100.0 | 358  (352-363) | 295  (242-347) | 2682  (2640-2723) | 2977  (2882-3070) |
| 12 | 2.1 | 1.9 | 98  (81-116) | 100.0 | 358  (352-363) | 590  (484-694) | 4076  (4013-4139) | 4666  (4497-4833) |
| 18 | 4 | 3.7 | 98  (81-116) | 100.0 | 358  (352-363) | 590  (484-694) | 7937  (7814-8060) | 8527  (8298-8754) |
| 24 | 2.3 | 2.2 | 98  (81-116) | 100.0 | 358  (352-363) | 590  (484-694) | 4720  (4646-4793) | 5309  (5130-5486) |
| 36 | 3.8 | 3.5 | 98  (81-116) | 100.0 | 358  (352-363) | 1180  (967-1387) | 15017  (14784-15249) | 16196  (15751-16636) |
| Sum | NA | NA | NA | NA | NA | NA | NA | 40974  (39757-42176) |

^*^：All HIV-positive participants were non-ART but all their sexual partners assumed to use PrEP every day; ^#^：According to the HIV prevalence among MSM, the HIV negative sexual partner adjusted by multiplying (1-0.0598); ART: antiretroviral therapy; $: U.S. dollars; PrEP: pre-exposure prophylaxis; NA: not applicable.

Table S9 Estimated cost of standard-ART plus partners’ PrEP treated scenarios over 36 months post-infection

| Month^*^ | Number of sexual partners (mean) | Adjusted negative sexual partners^#^ | ART cost per month ($) | Proportion unsuppressed (%) | PrEP cost per month ($) | ART cost within duration ($) | PrEP cost within duration ($) | Cost within duration ($) |
| --- | --- | --- | --- | --- | --- | --- | --- | --- |
| 3^a^ | 3.3 | 3.1 | 98  (81-116) | 100.0 | 358  (352-363) | 295  (242-347) | 3325  (3274-3377) | 3620  (3515-3723) |
| 6^a^ | 3.6 | 3.4 | 98  (81-116) | 96.4 | 358  (352-363) | 295  (242-347) | 3516  (3461-3570) | 3811  (3703-3917) |
| 12^a^ | 3.8 | 3.6 | 98  (81-116) | 100.0 | 358  (352-363) | 590  (484-693) | 7723  (7603-7842) | 8313  (8087-8536) |
| 18^a^ | 2.3 | 2.1 | 98  (81-116) | 100.0 | 358  (352-363) | 590  (484-693) | 4505  (4435-4575) | 5095  (4919-5268) |
| 23^a^ | 1.7 | 1.6 | 98  (81-116) | 85.7 | 358  (352-363) | 492  (403-578) | 2451  (2413-2489) | 2943  (2816-3067) |
| 24^b^ | 1.7 | 1.6 | 292  (263-322) | 85.7 | 358  (352-363) | 292  (263-322) | 490  (483-498) | 783  (745-820) |
| 36^b^ | 2.4 | 2.2 | 292  (263-322) | 62.5 | 358  (352-363) | 3510  (3153-3866) | 5899  (5808-5991) | 9409  (8961-9857) |
| Sum | NA | NA | NA | NA | NA | NA | NA | 33973  (32747-35187) |

^*^: All HIV-positive participants received ART based on the most current ART guideline from 2009-2016, and all sexual partners of HIV-positive participants who had unsuppressed viral load assumed to use PrEP every day; ^#^: The HIV negative sexual partners adjusted by multiplying by (1-0.0598); ^a^: No HIV-positive patients received ART and all sexual partners should take PrEP every day. the mean timing of ART initiation is 23 months after HIV infection. Cost within duration includes PrEP cost and ART cost; ^b^: HIV-positive patients with VL suppression just need ART, patients treated without VL suppression need both ART and sexual partners’ PrEP. Cost within duration includes all HIV-positive patients’ ART and sexual partners’ PrEP without VL suppression; ART: antiretroviral therapy; PrEP: pre-exposure prophylaxis; $: U.S. dollars; NA: not applicable.

Table S10 Estimated cost of early-ART plus partners’ PrEP scenarios over 36 months post-infection

| Month^*^ | Number of sexual partners (mean) | Adjusted negative sexual partners^#^ | ART cost per month ($) | Proportion unsuppressed (%) | PrEP cost per month ($) | ART cost within duration ($) | PrEP cost within duration ($) | Cost within duration ($) |
| --- | --- | --- | --- | --- | --- | --- | --- | --- |
| 3^a^ | 1.8 | 1.7 | 98  (81-116) | 98.8 | 358  (352-363) | 295  (242-347) | 1802  (1774-1829) | 2096  (2016-2176) |
| 6^b^ | 0.8 | 0.7 | 301  (269-333) | 87.1 | 358  (352-363) | 903  (808-998) | 654  (644-664) | 1557  (1452-1662) |
| 12^b^ | 0.6 | 0.6 | 301  (269-333) | 40.5 | 358  (352-363) | 1806  (1616-1996) | 521  (513-529) | 2328  (2130-2525) |
| 18^b^ | 1.2 | 1.1 | 292  (263-322) | 33.8 | 358  (352-363) | 1755  (1577-1933) | 798  (785-810) | 2552  (2362-2743) |
| 24^b^ | 1.5 | 1.4 | 292  (263-322) | 32.7 | 358  (352-363) | 1755  (1577-1933) | 982  (967-997) | 2737  (2543-2930) |
| 36^b^ | 0.8 | 0.8 | 292  (263-322) | 31.8 | 358  (352-363) | 3510  (3153-3866) | 1091  (1075-1108) | 4601  (4228-4974) |
| Sum | NA | NA | NA | NA | NA | NA | NA | 15872  (14730-17010) |

^*^: All HIV-positive participants received ART immediately, and all sexual partners of HIV-positive participants who had unsuppressed viral load assumed to use PrEP every day; ^#^: according to the HIV prevalence among MSM, the HIV negative sexual partner adjusted by multiplying by (1-0.0598); ^a^: No HIV-positive patients received ART and all sexual partners take PrEP every day. the mean timing of ART initiation is 3 months after HIV infection. Cost within duration includes PrEP cost and ART cost; ^b^: HIV-positive patients with VL suppression just need ART, patients treated without VL suppression need both ART and sexual partners’ PrEP. Cost within duration includes all HIV-positive patients’ ART and sexual partners’ PrEP without VL suppression; ART: antiretroviral therapy; PrEP: pre-exposure prophylaxis; $: U.S. dollars; NA: not applicable.
